# Primary Tumor Location and Lymphatic Spread in Papillary Thyroid Carcinoma

**DOI:** 10.1002/ohn.70474

**Published:** 2026-10-06

**Authors:** Isabelle Fournier, Mark E. Zafereo, Sarah Hamidi, Erich M. Sturgis, Steven G. Waguespack, Michelle D. Williams, S. Mohsen Hosseini, Mimi I. Hu, Paul H. Graham, Victoria Banuchi, Neil D. Gross, Miriam N. Lango, Jennifer R. Wang, Maria E. Cabanillas, Naifa L. Busaidy, Ramona Dadu, Priyanka Iyer, Steven I. Sherman, Sachin K. Gupta, Ryan P. Goepfert, Elizabeth G. Grubbs, Michael Kwon, Salmaan Ahmed, Anastasios Maniakas

**Affiliations:** ^1^ Department of Head & Neck Surgery The University of Texas MD Anderson Cancer Center Houston Texas USA; ^2^ Department of Endocrine Neoplasia and Hormonal Disorders The University of Texas MD Anderson Cancer Center Houston Texas USA; ^3^ Department of Otolaryngology–Head and Neck Surgery Baylor College of Medicine Houston Texas USA; ^4^ Department of Anatomical Pathology The University of Texas MD Anderson Cancer Center Houston Texas USA; ^5^ Department of Surgical Oncology The University of Texas MD Anderson Cancer Center Houston Texas USA; ^6^ Department of Neuroradiology The University of Texas MD Anderson Cancer Center Houston Texas USA

**Keywords:** locoregional metastasis, lymph node metastasis, neck dissection, papillary thyroid cancer, primary tumor location

## Abstract

**Objective:**

Papillary thyroid carcinoma (PTC) frequently metastasizes to cervical lymph nodes. Surgical planning is often based on the presumed central‐to‐lateral pattern of spread. The extent of central and lateral neck dissection remains controversial, with recommendations largely derived from small series. The study aimed to determine whether primary tumor location can guide the extent of neck dissection.

**Study Design:**

Single‐center retrospective cohort study.

**Setting:**

Quaternary care center.

**Methods:**

Patients with PTC undergoing primary thyroid surgery with a central and/or lateral neck dissection (CND and/or LND) between 1999 and 2023 were evaluated. Tumor location (superior, mid, inferior pole, or isthmus) was determined by ultrasound and confirmed on pathology. The primary outcome was pattern of nodal metastases by tumor location.

**Results:**

Among 1688 patients (median age 45 years; 67% female), 99% underwent CND and 45% LND. Central neck positivity was similar across superior (79%; 262/333), inferior (73%; 286/393), mid (74%; 626/849), and isthmic (76%; 71/94) tumors (*P* = .22). On multivariable logistic regression, central neck positivity did not differ significantly (superior OR: 1.28 [95% CI, 0.74‐2.23]; lower OR, 0.88 [95% CI, 0.52‐1.50]; mid OR, 0.79 [95% CI, 0.48‐1.32]; compared with isthmic. LND unadjusted and adjusted positivity rates were also comparable across primary tumor location. In patients undergoing LND, level III was most frequently involved (81%), followed by levels IV (75%), IIa (60%), Vb (36%), and IIb (9%) (*P* < .01). Lastly, superior pole tumors were significantly associated with ipsilateral level III involvement, compared with inferior and mid pole tumors (*P* < .01).

**Conclusions:**

In patients undergoing therapeutic neck dissection for PTC, primary tumor location did not predict nodal distribution and should not determine the extent of neck dissection.

Papillary thyroid carcinoma (PTC) is the most common thyroid malignancy, accounting for about 85%^1^ of cases. Clinical lymph node metastases (cN1) are present in up to one‐third of patients at presentation.^2^, ^3^ These include lymph nodes that appear abnormal either on preoperative imaging or upon physical examination (in clinic). Locoregional PTC has an excellent 5‐year overall survival (OS) rate of 99.3% (95% CI: 99‐95).^3^ However, it is associated with a higher rate of recurrence, estimated at 20%.^4^

Cervical metastases from PTC are traditionally believed to follow a stepwise pattern from the central compartment (levels VI and VII) to the lateral neck (levels II‐V). However, there is no consensus in the literature regarding the optimal extent of central (CND) and lateral neck dissection (LND) for PTC. Current guidelines on differentiated thyroid cancer (DTC)^5^ suggest the removal of all lymph nodes in levels IIa‐III‐IV‐Vb when lateral nodal disease is present (cN1b) (2025 ATA guidelines; recommendation 20). However, to minimize complications associated with LND—such as nerve injuries (spinal accessory, phrenic, brachial plexus, sympathetic chain, hypoglossal, vagus, greater auricular, and cervical sensory nerves), thoracic duct injury, hematoma, and infection—some experts advocate for a more selective approach,^6^, ^7^ challenging the ATA guidelines. Song et al^6^ found that selective LND (levels III and IV) could be considered for superior pole PTC patients with a single lymph node metastasis in levels III or IV. Conversely, McNamara et al^8^ reported that comprehensive LND (levels IIa‐III‐IV‐Vb) was associated with a higher regional control rate in the dissected neck, at 97.7%, compared with 89.4% with selective LND (levels III and IV) (*P* < .01). The 2025 ATA guidelines on DTC also recommend a therapeutic CND for positive central nodes (cN1a) (recommendation 20) and prophylactic unilateral or bilateral CND for positive lateral nodes (cN1b) or for T3N0‐T4N0 thyroid carcinoma (recommendation 19).^5^ The oncologic benefits of therapeutic CND are well established, whereas the role of prophylactic CND remains controversial.

The optimal extent of neck dissection in PTC therefore remains controversial, and the role of primary tumor location in guiding surgical planning is unclear. This study evaluates whether the location of the primary tumor within the thyroid (superior, mid, inferior pole, or isthmus) is associated with distinct patterns of central or lateral cervical lymph node metastasis. The aim is to determine whether primary tumor location can be used to tailor the extent of neck dissection.

## Methods

### Study Population

After obtaining approval from the MD Anderson Institutional Review Board (PA14‐1082), we conducted a retrospective chart review of adult patients with PTC who underwent primary thyroid surgery with neck dissection (central and/or lateral) at The University of Texas MD Anderson Cancer Center (MDACC) between October 1999 and August 2023. Inclusion criteria were patients with (1) histopathologic confirmation of PTC and (2) any type of thyroid surgery with neck dissection (n = 1688). Exclusion criteria were patients with (1) incomplete pathology reports due to missing information on primary tumor location (n = 76), (2) unavailable or incomplete preoperative ultrasound report (n = 140), (3) a discrepancy in tumor location between the postoperative pathology and preoperative ultrasound reports (n = 38), and (4) age <18 years old (n = 28) (Figure 1). Tumor location was defined as left lobe, right lobe, or isthmic, and further subdivided as superior, mid, or inferior when it was within a pole. Tumor location was determined by preoperative ultrasound and confirmed with final pathology. If the tumor was bilateral or multifocal, the largest tumor was selected as the primary of interest. Tumor staging was determined using the eighth edition of the American Joint Committee on Cancer (AJCC) staging system. The extent of neck dissection, as determined from operative notes, was classified as central and/or lateral and as unilateral and/or bilateral. Neck dissection surgical specimens were divided into levels by the surgeon in the operating room.

**Figure 1:**
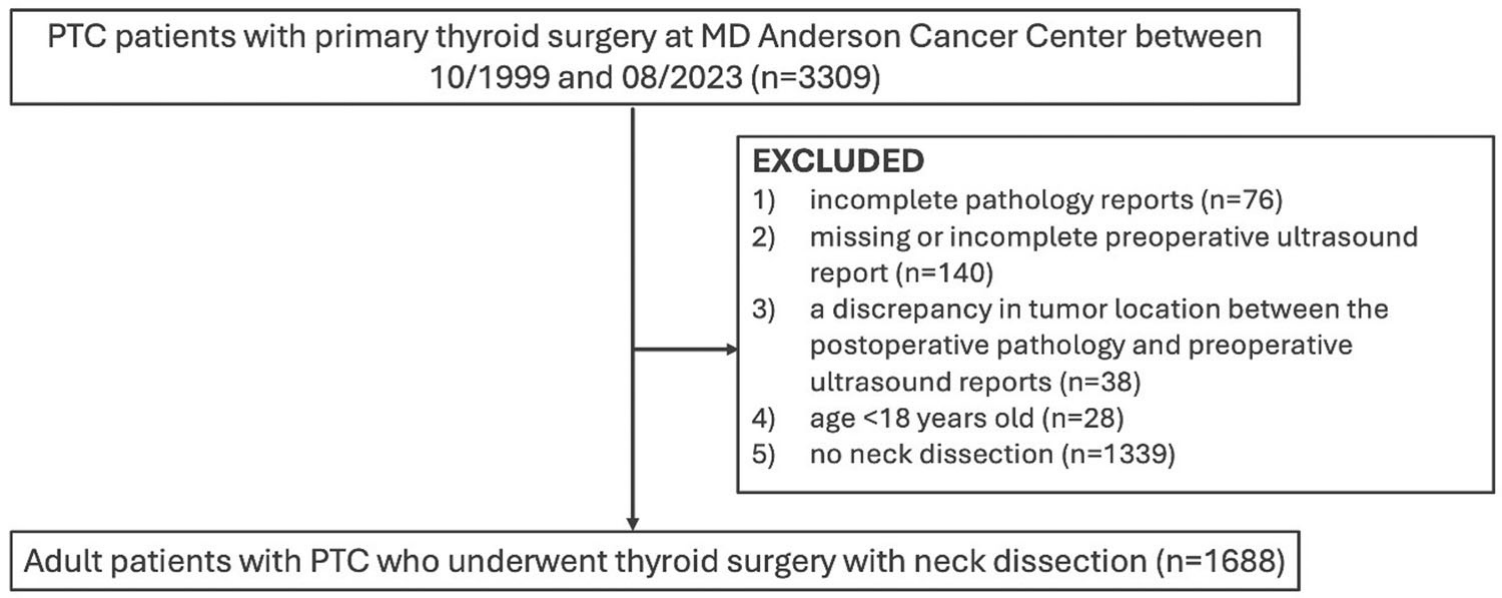
Patient selection flow diagram.

CND was defined as the removal of pretracheal, prelaryngeal (Delphian) and paratracheal lymph nodes (levels VI and VII). A CND was further characterized as either prophylactic (cN0) or therapeutic (cN1a). LND included levels IIa, IIb, III, IV, IVa, and IVb. LND was performed when there was clinical, radiologic, and/or histologic evidence of disease. Patients were included in LND group if at least one level in the lateral neck (determined from operative notes) was completely dissected. Gross extrathyroidal extension (ETE) was defined as any involvement of the strap muscles or major neck structures (subcutaneous tissues, larynx, RLN, esophagus, trachea, and prevertebral fascia) based on operative notes.

### Outcomes

This study's primary outcome was to evaluate the incidence and rate nodal disease by nodal level (IIa, IIb, III, IV, Va, Vb, VI) according to primary tumor location (superior, mid, or inferior poles). Demographics, preoperative ultrasonographic features, postoperative histopathological data, surgical history, and postoperative complications were collected from electronic medical records.

### Statistical Analysis

Continuous variables are presented as medians and ranges, while categorical variables are presented as frequencies and percentages. Between‐group associations were assessed using Pearson's chi‐square test for categorical variables and the Mann‐Whitney *U* test for continuous variables. Multivariate logistic regression was used to evaluate the association between primary tumor location and nodal metastasis location, adjusting for tumor size, gross ETE, and tumor stage. A *P* < .05 was indicative of statistical significance. Statistical analyses were performed with IBM SPSS Statistics for Macintosh, Version 29.0 (Released 2022. Armonk, NY: IBM Corp.).

## Results

### Patient Demographics

There were 1688 patients who underwent primary thyroid surgery with any neck dissection for PTC at MDACC during the study period, with demographics summarized in Table 1. Preoperative ultrasound identified 339 patients (20%) with upper pole tumors, 396 (23%) with lower pole tumors, 859 (51%) with mid pole tumors and 94 (6%) with isthmic tumors. Thirty‐one percent (n = 530/1688) and 44% (n = 735/1688) of patients had preoperative suspicious central and lateral lymph nodes on ultrasound, respectively. PTC subtypes included follicular (12%) versus not‐follicular (88%) which included classic, diffuse sclerosing, solid, insular, tall cell, columnar, cribriform, and warthin‐like subtypes. The median tumor size was 1.8 cm (range: 0.1‐11.9 cm). Forty‐five percent of patients had multifocal disease, 38% had lymphovascular invasion (LVI), and 18% had gross ETE. Central disease was found in 75% (1245/1669) of those undergoing CND, and in 95% (715/754) of those undergoing LND; 32% had extranodal extension (ENE).

**Table 1:** Demographic Characteristics of 1688 PTC Patients With Primary Thyroid Surgery With any Neck Dissection at UTMDACC 1999‐2023

Characteristics	All patients (n = 1688)
Sex—n (%)	
Male	566 (34)
Female	1122 (66)
Median age at diagnosis—yrs (range)	45 (18‐93)
pT—n (%)	
T1a	340 (20)
T1b	552 (33)
T2	386 (23)
T3a	106 (6)
T3b	182 (11)
T4a	120 (7)
T4b	2 (0)
pN—n (%)	
N0	383 (23)
N1a	590 (35)
N1b	715 (42)
M—n (%)	
Yes	74 (4)
No	1614 (96)
Multifocality	
Yes	764 (45)
No	924 (55)
*BRAF* V600E mutation—n (%)	
Negative	61 (25)
Positive	187 (75)
Unknown	1440 (−)
Thyroid surgery—n (%)	
Lobectomy	94 (6)
Thyroidectomy	1594 (94)
Central neck dissection—n (%)	
No	19 (1)
Unilateral	814 (48)
Bilateral	855 (51)
Lateral neck dissection—n (%)	
No	934 (55)
Unilateral	593 (35)
Bilateral	161 (10)

Abbreviations: M, metastasis; N, number; pN, pathologic nodal stage; pT, pathologic tumor stage; yrs, years.

### Stratification by Tumor Location

Demographic and pathologic characteristics by tumor location are detailed in Table 2. When comparing superior pole, inferior pole, mid‐pole, and isthmus tumors, gross ETE rates were 17%, 15%, 21%, 12% (*P* = .02), respectively, while median tumor sizes were 1.5 cm (range: 0.3‐6.0), 1.6 cm (range: 0.2‐7.0), 2 cm (range: 0.1‐11.9), and 1.6 cm (range: 0.1‐10.0) (*P* = <.01). Unadjusted central neck positivity rates did not differ significantly among tumor locations: 79% for the superior pole tumors (262/333), 73% for the inferior pole tumors (286/393), 74% for the mid‐pole tumors (626/849), and 76% for the isthmic tumors (71/94) (*P* = .22). On multivariable logistic regression adjusting for tumor size, gross ETE, and stage, the odds of central neck positivity did not differ significantly for superior‐pole tumors (OR: 1.28; 95% CI, 0.74‐2.23; *P* = .38), lower‐pole tumors (OR, 0.88; 95% CI, 0.52‐1.50; *P* = .64), or mid‐pole tumors (OR, 0.79; 95% CI, 0.48‐1.32; *P* = .37), compared with isthmic tumors.

**Table 2:** Clinical and Histopathological Characteristic Stratified by Tumor Location

	Superior pole (n = 339)	Mid‐pole (n = 859)	Inferior pole (n = 396)	Isthmus (n = 94)	*P*
Sex—n (%)					
Male	106 (31)	294 (34)	132 (33)	34 (36)	.74
Female	233 (69)	565 (66)	264 (67)	60 (64)	
Median age at diagnosis—yrs (range)	43 (18‐93)	45 (18‐88)	45 (18‐78)	46 (20‐82)	.23
pT—n (%)					
T1a	89 (26)	142 (17)	88 (22)	21 (22)	**.01**
T1b	119 (35)	244 (28)	150 (38)	39 (41)	
T2	69 (20)	211 (25)	87 (22)	19 (20)	
T3a	5 (1)	85 (10)	12 (3)	4 (4)	
T3b	38 (11)	105 (12)	32 (8)	7 (7)	
T4a	18 (5)	71 (8)	27 (7)	4 (4)	
T4b	1 (0)	1 (0)	0 (1)	0 (0)	
pN—n (%)					
N0	55 (16)	207 (24)	101 (26)	20 (21)	**.01**
N1a	82 (24)	288 (34)	174 (44)	46 (49)	
N1b	202 (60)	364 (42)	121 (30)	28 (30)	
M—n (%)					
Yes	329 (97)	809 (94)	387 (98)	89 (95)	**.02**
No	10 (3)	50 (6)	9 (2)	5 (5)	
Tumor size, median (range)	1.5 (0.3‐6.0)	2.0 (0.1‐11.9)	1.6 (0.2‐7.0)	1.6 (0.1‐10.0)	**.01**
Subtypes—n (%)					
PTC, follicular	33 (10)	116 (13)	47 (12)	6 (7)	.05
PTC, not‐follicular^a^	306 (90)	743 (87)	349 (88)	88 (93)	
Multifocal—n (%)					
No	195 (58)	471 (55)	208 (53)	50 (53)	.55
Yes	144 (42)	388 (45)	188 (47)	44 (47)	
LVI—n (%)					
No	206 (61)	512 (60)	260 (66)	61 (65)	.19
Yes	133 (39)	347 (40)	136 (34)	33 (35)	
ENE—n (%)					
No	233 (66)	578 (67)	278 (79)	63 (67)	.62
Yes	116 (34)	281 (33)	118 (30)	31 (33)	
Gross ETE—n (%)					
No	283 (83)	682 (79)	337 (85)	83 (88)	**.02**
Yes	56 (17)	177 (21)	59 (15)	11 (12)	

Bold values are statistically significant for P value.

Abbreviations: ENE, extranodal extension; ETE, extrathyroidal extension; LVI, lymphovascular invasion; n, number; PTC, papillary thyroid carcinoma; yrs, years.

^a^ Not‐follicular refers to classic, diffuse sclerosing, solid, insular, tall cell, columnar, cribriform, and Warthin‐like subtypes.

When evaluating LND positivity, unadjusted rates showed no significant difference, with 96% in superior pole tumors (202/210), 93% in inferior pole tumors (121/130), 95% in mid‐pole tumors (364/385), and 97% in isthmic tumors (28/29) (*P* = .59). On multivariable logistic regression adjusting for tumor size, gross ETE, and stage, the odds of lateral neck positivity did not differ significantly for superior‐pole tumors (OR, 1.03; 95% CI, 0.12‐8.73; *P* = .98), lower‐pole tumors (OR, 0.49; 95% CI, 0.06‐4.10; *P* = .51), or mid‐pole tumors (OR, 0.57; 95% CI, 0.07‐4.46; *P* = .59), compared with isthmic tumors.

In patients undergoing a LND (n = 754), the rate of positive disease was highest in level III, with 81% (338/417), followed by level IV at 75% (359/481), level IIa at 60% (240/401), level Vb at 36% (12/33), and level IIb at 9%(16/181) (*P* < .01). In level IIa, the median number of positive lymph nodes removed was 1 (range: 0‐6), with a median total of 9 lymph nodes removed (range: 0‐37). In level III, the median number of positive lymph nodes removed was 2 (range: 0‐13), with a median total of 9 lymph nodes removed (range: 1‐24). In level IV, the median number of positive lymph nodes removed was 1 (range: 0‐14), with a median total of 10 lymph nodes removed (range: 1‐35). Finally, in level Vb, the median number of positive lymph nodes removed was 0 (range: 0‐2), with a median total of 2 lymph nodes removed (range: 0‐10). When LND was performed based on preoperative evidence of disease, the incidence of central and lateral lymph node metastases by anatomic neck level for superior pole tumors (**A**), inferior pole tumors (**B**), and mid‐pole tumors (**C**) is detailed in Figure 2. Superior pole tumors metastasize more frequently to the ipsilateral level III compared to mid and lower pole tumors (*P* = <.01).

**Figure 2:**
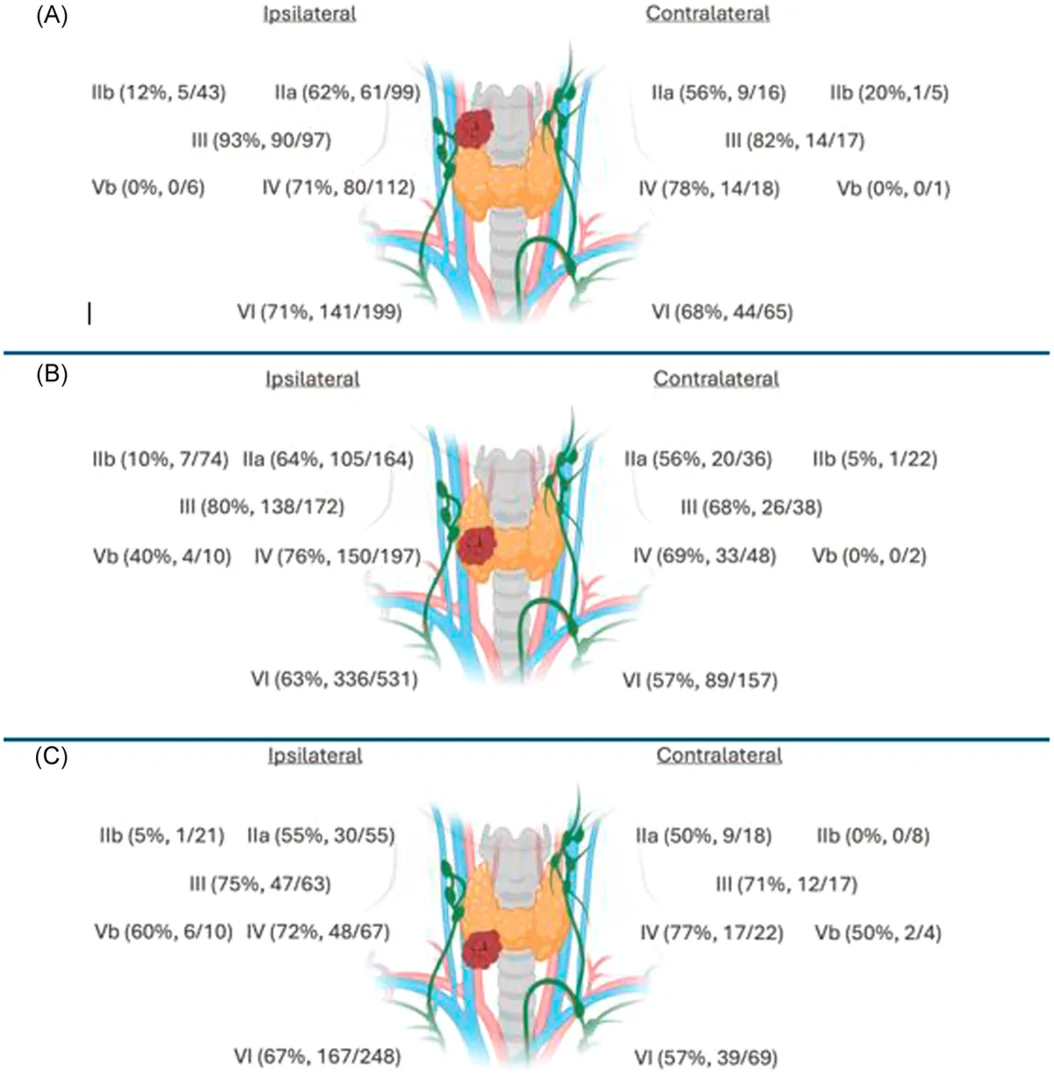
Incidence of ipsilateral and contralateral central and lateral lymph node metastasis for superior pole tumors (A), mid‐pole tumors (B) and inferior pole tumors (C) when neck dissection is indicated.

Regardless of the primary tumor location, among patients with metastasis in level IIa, 69 (87%) did not have involvement in level IIb, while 10 (13%) had confirmed metastasis in level IIb (*P* = .25). In patients with no disease in level IIa, ipsilateral IIb was positive in only 4 patients (7%). Additionally, among patients without metastasis in level III, 46 (67%) did not have metastasis in level IIa, whereas 23 (33%) had confirmed involvement in level IIa.

## Discussion

The goal of contemporary PTC management is to balance oncologic cure with minimization of treatment‐related morbidity. Over the last decade, there has been a shift toward de‐escalating/limiting the extent of surgery, particularly in the central and lateral neck, while maintaining excellent regional control. In this context, the possibility of tailoring the extent of neck dissection according to primary tumor location is appealing, but requires robust clinical evidence.

In this large, single‐institution contemporary series of 1688 patients undergoing primary surgery with a CND and/or LND, the location of the primary PTC did not meaningfully change the risk or distribution of cervical nodal metastases. Central neck positivity rates were uniformly high across superior, mid, inferior pole, and isthmic tumors, and lateral neck positivity rates were similarly comparable. These findings suggest that the cranio‐caudal position of the primary tumor should not be used as a surrogate for overall nodal risk or as a basis to omit central or certain lateral compartments from a therapeutic dissection.

Within the lateral neck, the pattern of involvement was also broadly consistent regardless of primary tumor location. Levels III and IV were most frequently involved, followed by IIa and Vb, while IIb was less frequently positive. Superior pole tumors were more often associated with ipsilateral level III disease than inferior and mid pole tumors, although this difference did not translate to a distinct, clinically useful pattern that would justify restricting dissection to selected levels. For example, inferior pole tumors still demonstrated substantial rates of level IIa involvement, arguing against limiting dissection to levels III and IV. The overall distribution of disease across levels IIa, III, IV, and Vb in this cohort supports the use of a systematic IIa‐Vb dissection when a LND is indicated.

Our findings also refine the role of level IIb dissection. In the presence of disease in level IIa, IIb was involved in 13%, which is not insignificant. Given the increased risk of spinal accessory nerve injury associated with dissecting level IIb, these results support reserving level IIb dissection for cases with radiologic or intraoperative evidence of disease, rather than routine dissection. However, re‐operation in level IIb places the accessory nerve at higher risk, and prophylactic level IIb dissection is preferred by some thyroid cancer surgeons, especially when level IIa is involved. Conversely, even with a negative level III, level IIa should systematically be dissected as it is still associated with a 33% chance of positive disease.

Understanding and mapping the location of the primary tumor in a preoperative setting may still have clinical implications. For example, during preoperative imaging, it is important to gather information on the anterior‐posterior position of the tumor, as posterior tumors carry a higher risk of RLN injury. Posterior tumors are also associated with a higher risk stratification when the margin is positive as per the 2025 ATA guidelines.^5^ Additionally, understanding the cranio‐caudal location of the thyroid tumor can help predict ETE, as our findings demonstrate that mid‐pole tumors are associated with higher rates of ETE and larger median tumor size. We believe mid‐pole tumors are generally larger at the time of diagnosis due to the ability of the thyroid to conceal itself deep in the neck. As such, as these grow larger prior to detection, they may have higher rates of ETE.

### Limitations

Limitations of this study include its retrospective design and may introduce the possibility of selection bias, while surgical and pathologic practices may have evolved over the 24‐year study period. Furthermore, not all LND specimens were divided by level, which led to smaller denominators for some level‐specific analyses. In cases where the tumor was bilateral or multifocal, the largest tumor was designated as the index lesion, which may not fully capture the complexity of multifocal disease. Another limitation of this study is the potential imprecision of tumor localization in patients with large tumors. While our patient population appears to represent the general demographics of PTC, bias related to referral to a quaternary care center is present. Another limitation is the 24‐year study period, during which imaging has changed and improved substantially over time, potentially influencing the results. Nonetheless, the size of the cohort and the detailed mapping of nodal involvement provide robust, contemporary evidence to inform surgical decision‐making.

## Conclusion

In one of the largest cohorts of patients undergoing primary thyroid surgery with neck dissection for PTC, primary tumor location within the thyroid (superior, mid, inferior pole, or isthmus) did not significantly alter the likelihood of central or lateral cervical lymph node metastasis. Therefore, tumor location alone should not be used to limit the extent of therapeutic neck dissection. When a LND is indicated based on clinical or radiological evidence of disease, these data support a systematic dissection from levels IIa through Vb to achieve adequate regional control.

## Author Contributions

**Isabelle Fournier**, data curation, conceptualization, methodology, formal analysis, writing—original draft preparation, writing—reviewing and editing; **Mark E. Zafereo**, writing—reviewing and editing; **Sarah Hamidi**, writing—reviewing and editing; **Erich M. Sturgis**, writing—reviewing and editing; **Steven G. Waguespack**, writing—reviewing and editing; **Michelle D. Williams**, writing—reviewing and editing; **S. Mohsen Hosseini**, writing—reviewing and editing; **Mimi I. Hu**, writing—reviewing and editing; **Paul H. Graham**, writing— reviewing and editing; **Victoria Banuchi**, writing—reviewing and editing; **Neil D. Gross**, writing—reviewing and editing; **Miriam N. Lango**, writing—reviewing and editing; **Jennifer R. Wang**, writing—reviewing and editing; **Maria E. Cabanillas**, writing—reviewing and editing; **Naifa L. Busaidy**, writing—reviewing and editing; **Ramona Dadu**, writing—reviewing and editing; **Priyanka Iyer**, writing—reviewing and editing; **Steven I. Sherman**, writing—reviewing and editing; **Sachin K. Gupta**, data curation; **Ryan P. Goepfert**, writing— reviewing and editing; **Elizabeth G. Grubbs**, writing—reviewing and editing; **Michael Kwon**, radiological evaluation of cohort; **Salmaan Ahmed**, radiological evaluation of cohort; **Anastasios Maniakas**, conceptualization, methodology, formal analysis, writing—original draft preparation, writing—reviewing and editing.

## Disclosures

### Competing interests

None.

### Funding source

None.
